# Restoration of plakoglobin expression in bladder carcinoma cell lines suppresses cell migration and tumorigenic potential

**DOI:** 10.1038/sj.bjc.6602651

**Published:** 2005-06-07

**Authors:** K M Rieger-Christ, L Ng, R S Hanley, O Durrani, H Ma, A S Yee, J A Libertino, I C Summerhayes

**Affiliations:** 1 Cell and Molecular Biology Laboratory, Robert E Wise MD Research and Education Institute, 31 Mall Road, Burlington, MA 01805, USA; 2 Department of Urology, Lahey Clinic, 41 Mall Road, Burlington, MA 01805, USA; 3 Tufts University School of Medicine, 136 Harrison Avenue, Boston, USA

**Keywords:** plakoglobin, bladder cancer, desmosome, signalling

## Abstract

The reduction or loss of plakoglobin expression in late-stage bladder cancer has been correlated with poor survival where upregulation of this catenin member by histone deacetylase inhibitors has been shown to accompany tumour suppression in an *in vivo* model. In this study, we directly addressed the question of the role of plakoglobin in bladder tumorigenesis following restoration, or knockdown of expression in bladder carcinoma cell lines. Restoration of plakoglobin expression resulted in a reduction in migration and suppression of tumorigenic potential *in vivo*. Immunocytochemistry revealed cytoplasmic and membranous localisation of plakoglobin in transfectants with <1% of cells displaying detectable nuclear localisation of plakoglobin. siRNA knockdown experiments targeting plakoglobin, revealed enhanced migration in all cell lines in the presence and absence of E-cadherin expression. In bladder cell lines expressing low levels of plakoglobin and desmoglein-2, elevated levels of desmoglein-2 were detected following restoration of plakoglobin expression in transfected cell lines. Analysis of wnt signalling revealed no activation event associated with plakoglobin expression in the bladder model. These results show that plakoglobin acts as a tumour suppressor gene in bladder carcinoma cells and the silencing of plakoglobin gene expression in late-stage bladder cancer is a primary event in tumour progression.

Loss or altered expression of cadherin or catenins in neoplastic progression has been a frequent finding in a variety of human cancers (Gee *et al*, 1998; Bukholm *et al*, 2000; Bussemakers *et al*, 2000; Hajra and Fearon, 2002), where altered E-cadherin expression has featured most commonly as an indicator of poor prognosis in bladder cancer (Shimazui *et al*, 1996; Mialhe *et al*, 1997; Shariat *et al*, 2001). Loss of expression of the invasive suppressor gene E-cadherin in bladder carcinoma cell lines has been associated with novel expression of the invasive promoter gene N-cadherin (Gee *et al*, 1998). Downregulation of plakoglobin accompanies such events in bladder tumorigenesis although we do not know whether changes in plakoglobin expression contribute to tumour progression. Recently, we have shown that exposure of late-stage bladder carcinoma cells to histone deacetylase inhibitors upregulates plakoglobin expression and suppresses the tumorigenic potential (Canes *et al*, 2005). However, from these experiments we cannot assess the contribution of plakoglobin to tumour suppression since multiple genes are modulated following exposure to this class of agent.

Numerous studies report that altered expression of individual catenins is correlated to prognosis (Shimazui *et al*, 1996; Mialhe *et al*, 1997; Morita *et al*, 1999) with alterations in plakoglobin expression linked to clinical outcome in different tumour types (Pantel *et al*, 1998; Amitay *et al*, 2001; Papergerakis *et al*, 2004). In bladder cancer, loss or reduced plakoglobin expression has been correlated with poor survival (Shimazui *et al*, 1996; Syrigos *et al*, 1998). Despite the growing number of correlative studies, the possible role or mechanism by which each individual member of the cadherin–catenin complex may contribute to neoplastic progression is not fully understood.

Plakoglobin, also referred to as *γ*-catenin, is a structural and functional homolog of *β*-catenin and Armadillo, both of which serve adhesion and signalling roles in development (Ben-Ze'ev and Geiger, 1998; Bierkamp *et al*, 1999; Kolligs *et al*, 2000). However, plakoglobin differs from *β*-catenin by interaction with the desmosomal cadherins (Marcozzi *et al*, 1998; Bierkamp *et al*, 1999) where it is an indispensable structural element of the desmosome.

Increased *β*-catenin levels have been shown to result in *β*-catenin binding to transcription factors of the Tcf/Lef family accompanied by increased tumorigenicity, indicating that *β*-catenin has oncogenic potential (Peifer, 1997). In contrast, several groups have reported that plakoglobin acts as a tumour suppressor gene. This was initially inferred from studies showing loss of heterozygosity of the plakoglobin gene in certain types of tumours (Aberle *et al*, 1995), reduced plakoglobin expression in different tumour types (Sommers *et al*, 1994; Pantel *et al*, 1998; Syrigos *et al*, 1998) and the demonstration that plakoglobin overexpression can suppress the tumorigenicity of mouse and human cells (Simcha *et al*, 1996). Recently, however, several reports have suggested that plakoglobin can have oncogenic properties in specific tissues. This hypothesis originated from the observation that plakoglobin expression correlated positively with the grade of hepatocellular carcinomas, being the highest in poorly differentiated tumors. A positive correlation was also found between elevated expression of plakoglobin and vascular invasion (Endo *et al*, 2000). More direct evidence was presented by Kolligs *et al* (2000), who demonstrated that overexpression of wild-type plakoglobin elicits neoplastic transformation in a rat kidney epithelial cell line.

In this study we report restoration of plakoglobin expression in late-stage bladder carcinoma cells suppresses migration and *in vivo* tumorigenicity. In the bladder carcinoma model, where downregulation of plakoglobin appears to accompany loss of E-cadherin expression in tumour progression, plakoglobin plays a tumour suppressor role.

## MATERIALS AND METHODS

### Cell culture

Human bladder cell line RT4, EJ, 5637, RT112, HT1376, HU456, KK47, UM-UC-3, SCaBER, J82, T24, TCCSUP, MGHU1, CUBIII, PSI, HT1197 (ATCC) and BC16 (kindly provided by Dr C Reznikoff, University of Wisconsin) were maintained in Dulbecco's Modified Eagle's Medium (DMEM) supplemented with 7.5% foetal bovine serum and penicillin/streptomycin.

### Transfection

EJ and J82 bladder carcinoma cell lines were plated at 3 × 10^5^ cells per 60 mm dish 24 h before lipofection. The plakoglobin consruct (generous gift from Dr Ben-Ze'ev) or the neomycin resistance gene was introduced into cells using the lipofectin reagent (Gibco BRL, Gaithersburg, MD, USA) Following incubation, cells were washed and maintained in standard medium. At 48 h post-transfection, cells were split into neomycin containing medium to select for successfully transfected colonies. Individual colonies screened for drug selection were ring-cloned 2 weeks later.

### Antibodies

The following antibodies were used in the study: N-cadherin clone 13A9 (1 : 200 kindly provided by Dr M Wheelock, Eppley Cancer Center, WI, USA), plakoglobin (1 : 1000), β-catenin (1 : 1000), (Transduction Laboratories, Lexington, KY, USA), E-cadherin (1 : 500 Zymed Laboratories, South San Francisco, CA, USA) and *β*-actin (1 : 1000 Sigma, St Louis, MD, USA).

### Western blot analysis

Subconfluent dishes of cells were washed in phosphate-buffered saline (PBS) followed by lysis in hot sample buffer (0.08 M Tris, pH 6.8; 0.07 M SDS, 10% glycerol, 0.001% bromophenol blue and 1 mM CaCl_2_) and sheared through a 26-gauge needle. Lysates were then assayed for protein concentration using the BSA method (Pierce, Rockford, IL, USA). After determination of protein content, *β*-mercaptoethanol (1%) was added to each sample. Samples were boiled for 5 min and protein was loaded in each lane of a 7.5% polyacrylamide gel. Proteins were transferred overnight onto nitrocellulose. Membranes were blocked in 10% milk in TBS with 0.05% Tween (TBST) and placed on primary antibody overnight at 4°C. Blots were washed in TBST, three times for 15 min each, and secondary antibody linked to horseradish peroxidase was incubated with the blots for 60 min at room temperature. Blots were then washed as described above and developed with an ECL kit (Amersham, Arlington Heights, IL, USA).

### siRNA transfection

The siRNA target sequence AGTCGGCCATTGTGCATCT (start – nucleotide 490, Dharmacon siDesign Center, Dharmacon RNA Technologies, Lafayette, CO, USA) was selected based on a lack of homology with other catenin members and localisation at the 5′ end of the gene. Target cells were seeded at 4 × 10^4^ cells per well in a 24-well plate in media containing 10% foetal bovine serum. Transfection of the RNA oligonucleotide and a scrambled control sequence was performed using Oligofectamine (Invitrogen, Carlsbad, CA, USA) to result in a final RNA concentration of 200 nM. The cells were harvested 48 h post-transfection and used for either Western blot analysis or migration assays.

### Migration and invasion assays

*In vitro* cell migration and invasion assays were carried out using modified Boyden chambers consisting of Transwell (Corning Costar Corp., Cambridge, MA, USA) membrane filter inserts (pore size 8 *μ*m) in 24-well tissue culture plates. For migration assays, fibronectin (20 ug ml^−1^) was used as the chemoattractant. For invasion assays the upper surfaces of the membranes were coated with Matrigel (Collaborative Biomedical Products, Bedford, MA, USA) and placed into 24-well tissue culture plates containing 600 *μ*l of NIH/3T3 conditioned media (experimental) or plain DMEM (control). In both assays, cells (1 × 10^5^) were added to each Transwell chamber and allowed to invade toward the underside of the membrane for 8 (J82) or 16 h (EJ) at 37°C. Cells that passed through the membrane were fixed in methanol, stained with crystal violet and counted under a light microscope. Statistical analysis was performed by a Student's *t*-test, where *P*<0.05 was considered significant.

### Growth in Agar

Agar was made by combining one part 0.9% agarose in DMEM with two parts DMEM/20% foetal calf serum. A measure of 3 ml aliquots were plated into 60 mm bacteriologic dishes and allowed to gel. Agar top layer was made by combining one part 1.5% agarose in DMEM with two parts DMEM/12% foetal calf serum and sufficient cells to achieve a final concentration of either 1 × 10^5^ or 5 × 10^5^ cells per 60 mm dish. After solutions had solidified at room temperature, dishes were returned to the incubator and fed biweekly by the addition of 1.5 ml of agar top.

### Tumorigenicity assays

Subconfluent dishes of tranfectant and parental cell lines were harvested, washed in PBS followed by subcutaneous inoculation into nu/nu mice (1 × 10^6^ cells inoculum^−1^) using a 26-gauge needle. Weekly checks for tumour growth and animal health status were performed. Tumours were measured using calipers and tumour volume was calculated using the following formula: tumour volume=length × width × height × *π*/6. Animal experiments were carried out under the regulations set by the Institutional Animal Care and Use Committee guidelines.

### Immunocytochemistry

Cells were grown on glass slides, washed with PBS and fixed in 3.7% formaldehyde for 15 min at room temperature. Cells were then rinsed in three changes of PBS and permeabilised in 0.5% Triton X-100 in PBS. Following three washes in PBS, cells were stained for plakoglobin (Transduction Laboratories, Lexington, KY, USA) using an automated immunohistochemical processor (Model 320; Ventana Medical Systems, Tucson, AZ, USA).

### Wnt signalling

Cells were transfected with either 0.8 *μ*g TOPFLASH or FOPFLASH, 0.4 *μ*g *β*-Gal and 0.8 *μ*g control, *β*-catenin or plakoglobin plasmid. Cells were harvested 48 h post-transfection and luciferase and *β*-Gal activity were measured. Results were scored as luciferase activity normalised to *β*-Gal activity.

## RESULTS

### Cadherin and plakoglobin expression in bladder carcinoma cell lines

**Figure 1:**
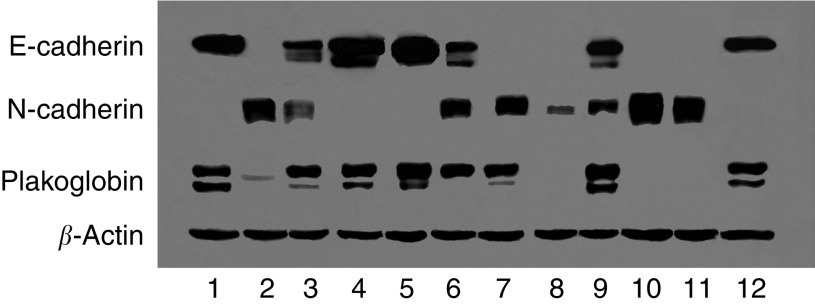
Western blot analysis showing endogenous levels of E-cadherin, N-cadherin, plakoglobin and *β*-actin expression in a representative panel of bladder carcinoma cell lines. Lane 1, RT4; lane 2, EJ; lane 3, 5637, lane 4, RT112; lane 5, HT1376; lane 6, HU456; lane 7, KK47; lane 8, UM-UC-3; lane 9, SCaBER; lane 10, J82; lane 11, TCCSUP; lane 12, BC16. Note the cell lines TCCSUP, J82, UM-UC-3 and EJ express N-cadherin in the absence of E-cadherin and display correspondingly low levels of plakoglobin.

The expression profile of classic cadherins within a series of bladder carcinoma cell lines has been described (Gee *et al*, 1998). Figure 1 shows total cell lysates from a representative bladder carcinoma cell panel probed in Western blot analysis. This panel includes bladder cell lines expressing E-cadherin in the absence of N-cadherin, E-cadherin in the presence of N-cadherin and N-cadherin in the absence of E-cadherin. It is of note that, with the exception of KK47, cell lines lacking E-cadherin expression display minimal plakoglobin expression. These results are similar to observations made in other human carcinoma cell lines (Bussemakers *et al*, 2000; Winn *et al*, 2002) and suggest a link between the presence of E-cadherin and the level of plakoglobin expression.

### Establishment and characterisation of plakoglobin transfectants

**Figure 2:**
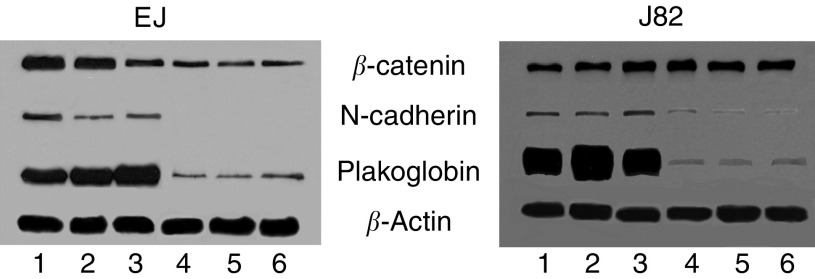
Western blot analysis showing EJ and J82 transfectants probed for expression of *β*-catenin, N-cadherin, plakoglobin and *β*-actin. Lanes 1–3 in each panel represent three clones expressing high levels of plakoglobin following transfection. Lanes 4–6 represent three control clones harboring the neomycin-resistance gene.

**Figure 3:**
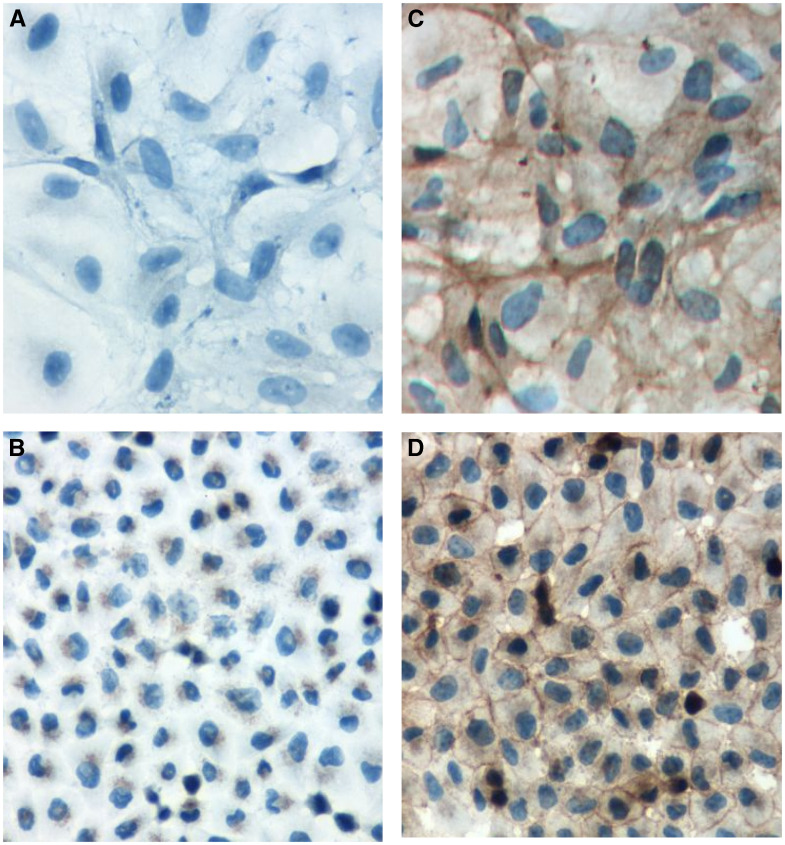
Parental cell lines (**A**) J82 and (**B**) EJ displayed no detectable staining of plakoglobin in immunohistochemistry. In J82 transfectants (**C**) cytoplasmic with limited membranous plakoglobin was observed. Less than 1% of transfectants displayed nuclear localization of plakoglobin. In EJ transfectants (**D**) cytoplasmic and membranous localization of plakoglobin was recorded with no detectable nuclear plakoglobin.

Late-stage events in bladder tumorigenesis include loss of E-cadherin expression and downregulation of plakoglobin. Although it has been shown that E-cadherin is an invasive suppression gene, it is not known whether the reduction in plakoglobin expression observed in these carcinoma cells has a primary or secondary role to play in tumour progression. To address this question we selected representative bladder cell lines that lack E-cadherin and have low plakoglobin expression levels (EJ and J82) as recipients in transfection. In each case, our goal was to restore plakoglobin expression to levels recorded in earlier stages of tumour progression. Restoration of plakoglobin expression in bladder cell lines EJ and J82 was achieved following lipofection and confirmed in Western blot analysis (Figure 2). Three EJ and J82 clones were selected following limiting dilution, identified as expressing levels of plakoglobin comparable to that recorded in E-cadherin cell lines (Figure 1). In EJ and J82 cell lines, the level of N-cadherin expression was increased in plakoglobin transfectants as compared to neomycin controls. Immunocytochemical evaluation of each clone revealed cytoplasmic and membranous localisation of plakoglobin (Figure 3) with <1% of cells displaying nuclear localisation of plakoglobin. No difference in growth rate was recorded between EJ and J82 cells overexpressing plakoglobin and neomycin control transfectants. The cloning efficiency of the EJ control and plakoglobin transfectants in soft agar was 28±1.5 and 11±1%, respectively. The bladder cell line J82 and the plakoglobin transfectants did not form colonies in this assay.

### Migration and invasion assays

**Figure 4:**
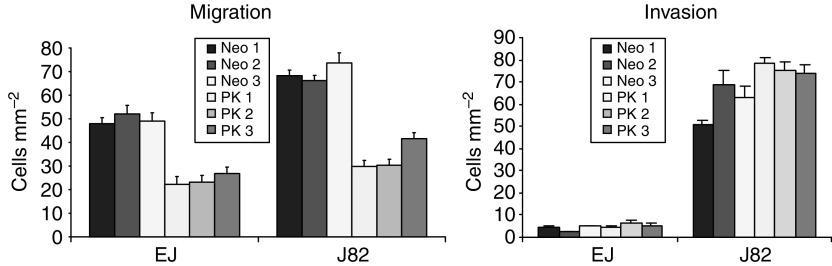
*In vitro* migration and invasion assay results showing decreased migration of plakoglobin transfectants with no significant alteration in the invasive capacity as compared to neomycin controls.

We initially evaluated the effect of forced plakoglobin expression on the invasion and migration potential of transfected cells. Using standard *in vitro* assays, EJ and J82 plakoglobin transfectants were tested in migration assays. Figure 4 shows results from a representative assay demonstrating a significant reduction in migration in both EJ and J82 plakoglobin transfectants. Using the same cell lines in invasion assays, no significant change in the invasive capacity of the cells was detected in repeated assays (Figure 4).

### Downregulation of plakoglobin using siRNA

**Figure 5:**
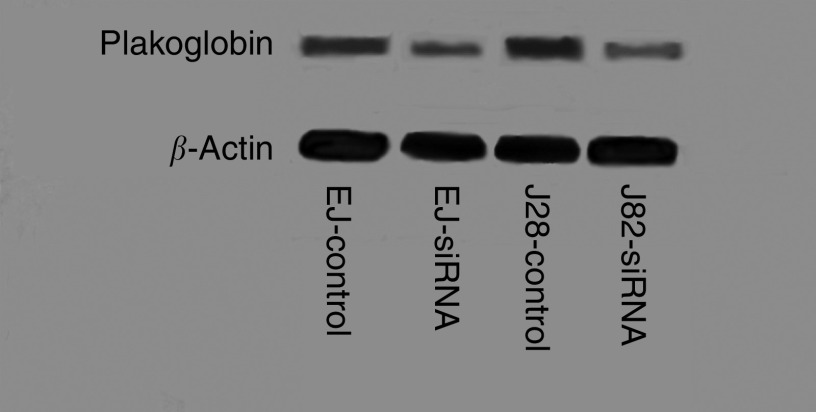
siRNA directed against plakoglobin downregulates protein expression. EJ and J82 cells were transfected with siRNA directed against plakoglobin or control transfected with a scrambled siRNA. Cells were lysed 48-h post-transfection and Western blots were probed with anti-plakoglobin or anti-*β*-actin antibody.

**Figure 6:**
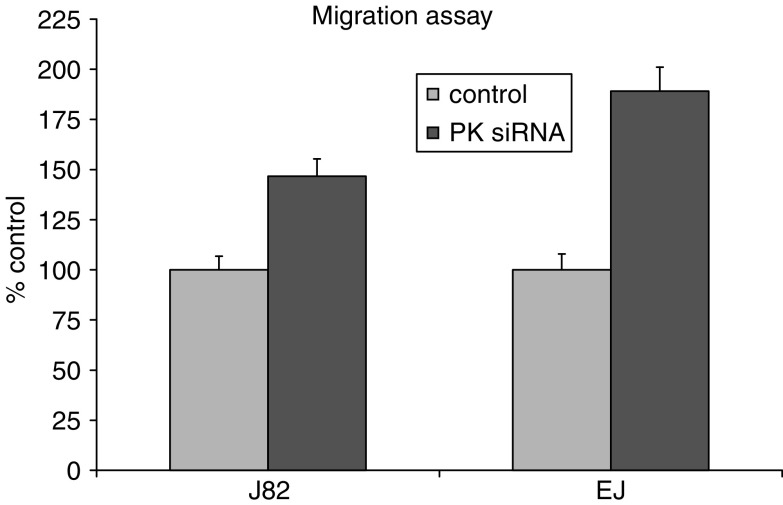
Migration assay of EJ and J82 cells transfected with plakoglobin siRNA or control transfected controls harbouring scrambled siRNA sequence. The cells transfected with siRNA directed against plakoglobin displayed a statistically significant increase in migration capacity compared to the mock-transfected controls. Numbers are expressed as percent control where mock-transfectants were considered 100%.

If plakoglobin has a role to play in cell migration, downregulation of this catenin should promote migrational activity in bladder carcinoma cells. Figure 5 shows a Western blot analysis of siRNA experimental and control transfectants demonstrating downregulation of plakoglobin in EJ and J82 cells transfected with plakoglobin siRNA. Figure 6 shows enhanced migration of both cell lines following downregulation of plakoglobin expression, a result consistent with and reciprocal to, the inhibition of migration recorded following restoration of plakoglobin expression. Harvesting parallel cultures of transfectants at the end of the migration assay revealed continued downregulation of plakoglobin at the 72-h time point.

To address the question as to whether reduction of plakoglobin expression would alter migration in bladder carcinoma cells expressing E-cadherin, we performed the same experiment on CUBIII and HU456 bladder carcinoma cell lines that express E-cadherin. siRNA knockdown of plakoglobin increased the migratory potential in both CUBIII and HU456 cells by 145.9±8.4 and 185.2±22%, respectively.

### Tumorigenic potential of plakoglobin transfectants

Parental cell line J82 is nontumorigenic when implanted subcutaneously into nude mice. At 6 weeks, no tumour formation was recorded in the three J82 plakoglobin transfectants tested *in vivo* (0 out of 12). After the same time period EJ neo-transfectants produced large tumours at 11 out of 12 sites with EJ plakoglobin transfectants generating tumours in three out of 12 sites. Tumours arising from EJ neo-controls recorded average tumour volumes >5 times that recorded for EJ plakoglobin transfectant tumours.

### Relationship between expression levels of plakoglobin and desmosomal proteins

**Figure 7:**
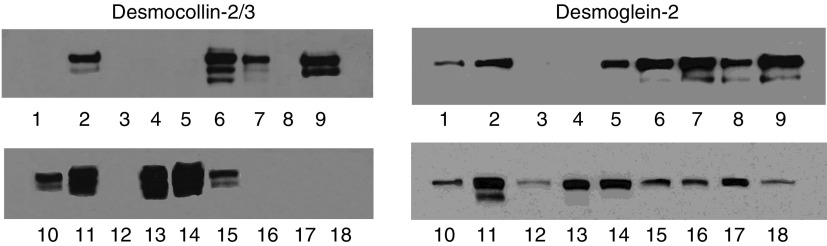
Western blot analysis showing desmocollin-2/3 and desmoglein-2 expression in a panel of bladder carcinoma cell lines. Note that cell lines expressing reduced levels of plakoglobin (Figure 1) also show reduced expression of desmoglein and desmocollin. Lane 1, EJ; lane 2, 5637; lane 3, J82, lane 4, TCCSUP; lane 5, T24; lane 6, SCaBER; lane 7, RT112; lane 8, RT4; lane 9, PSI; lane 10, BC16.1; lane 11, CUBIII; lane 12, EJ; lane 13, HT1197; lane 14, HT1376; lane 15, HU456; lane 16, UM-UC-3; lane 17, KK47; lane 18, MGHU-1. Cell lysate from the EJ cell line was included in all gels.

**Figure 8:**
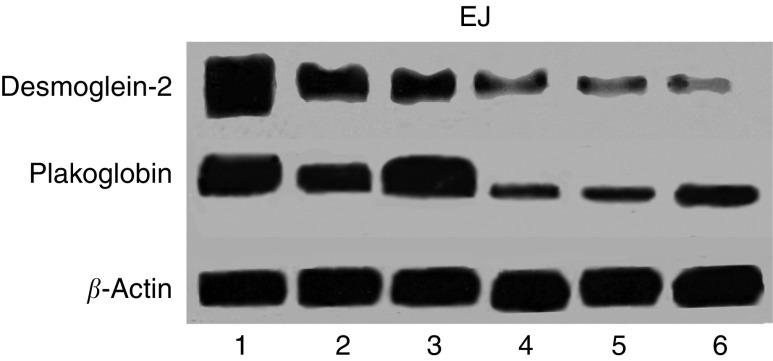
Western blot analysis showing total cell lysates from EJ plakoglobin transfectant clones (lanes 1–3) and neomycin control cell lysates (lanes 4–6) probed for desmoglein-2, plakoglobin and *β*-actin.

Plakoglobin is not only found in the cadherin complex but exists as an integral component of the desmosomal complex. We examined the constitutive expression levels of desmocollin-2 and desmoglein-2 in the panel of bladder carcinoma cell lines. Figure 7 shows an absence or reduced expression of desmocollin-2 and desmoglein-2 expression in cells lines in which plakoglobin levels were low. To determine the relationship between the levels of desmosomal proteins and plakoglobin, we analysed the expression levels of desmoglein-2 in the control and plakoglobin transfectants of each bladder carcinoma cell recipient. As shown in Figure 8, the expression levels of desmoglein-2 increased in EJ plakoglobin transfectants but remained undetected in J82 control and plakoglobin transfectants (data not shown).

### Signalling pathway(s) following forced plakoglobin expression

APC is reported to regulate both *β*- and *γ*-catenin where the oncogenic action of *β*-catenin is dependent on Tcf/Lef function. In addition, the oncogenic action of *γ*-catenin in kidney epithelial cells was linked to elevated c-myc expression (Kolligs *et al*, 2000). We have probed lysates from parental and transfected cells to establish the levels of c-myc expression and have found no alteration in the expression level of this nuclear protein in either EJ or J82 plakoglobin transfectants. We also probed lysates for MAP kinase and AKT expression and activation, demonstrating unchanged levels of both in parental and transfected cell lines (data not shown).

Although *β*-catenin's role in wnt signalling has been well established, the role of plakoglobin in this pathway is less clear. To determine basal levels of wnt signalling in EJ and J82 cells, we scored the activation of Tcf/Lef-dependent transcription using the activity of the TOPFLASH reporter as an end point. Both cell lines exhibited low levels of wnt activity (data not shown). When *β*-catenin was included in the assay J82 showed no significant wnt activation (Top+*β*-cat/Top=1.9), whereas EJ showed a modest increase (Top+*β*-cat/Top=13). Hence, we proceeded to determine whether plakoglobin would effect wnt signalling in the EJ cell line. Although plakoglobin decreased TOPFLASH activity (Top+PK/Top=0.26), it also affected FOPFLASH (Fop+PK/Fop=0.52). Since the FOPFLASH reporter contains mutant Tcf/Lef sites, these results suggest that plakoglobin does not mediate its effects through the wnt pathway in this model.

## DISCUSSION

Plakoglobin has been reported to act both as an oncogene and a tumour suppressor gene depending on the cell type (Simcha *et al*, 1996; Kolligs *et al*, 2000; Winn *et al*, 2002). Although a role for plakoglobin in bladder cancer has not been established, reduced plakoglobin expression has been linked to poor prognosis in patients with bladder carcinoma (Syrigos *et al*, 1998).

To directly investigate the role of plakoglobin in bladder tumorigenesis, we transfected plakoglobin into bladder carcinoma cells expressing low levels of endogenous protein. Using standard *in vitro* migration and invasion assays, we demonstrated inhibition of migratory potential with no significant alteration recorded in the invasive capacity of the plakoglobin transfectants.

In reciprocal experiments, where plakoglobin expression was knocked down using siRNA, we demonstrated enhanced migration. This was observed in bladder carcinoma cell lines expressing either E- or N-cadherin. Loss of plakoglobin from the E-cadherin complex likely compromises its functionality resulting in enhanced migration. In N-cadherin expressing cells, the reduction in minimal levels of plakoglobin also promoted migration. These results suggest that reduced expression of plakoglobin in bladder tumorigenesis is a significant event contributing to increased cell migration, one aspect of the invasive phenotype.

The J82 bladder carcinoma cell line was established from a late-stage tumour and displays no tumorigenic potential following subcutaneous implantation in nude mice (Masters *et al*, 1986). The restoration of plakoglobin expression did not result in the acquisition of tumorigenic potential in this cell line demonstrating a lack of oncogenic action of plakoglobin in this model. In contrast, the EJ cell line is tumorigenic in this *in vivo* assay, and forced expression of plakoglobin in transfected cells resulted in tumour suppression, further supporting the concept that downregulation of plakoglobin is a significant event associated with bladder tumorigenesis.

Plakoglobin not only associates with cadherins but also exists as an integral part of the desmosome complex. At the intracellular aspect, desmogleins (Dsg) and desmocollins (Dsc) bind plakoglobin, which in turn binds directly to the N-terminus of desmoplakin where the latter bridges the desmosomal complex to the intermediate filaments. Although the different isoforms of Dsg and Dsc can be found differentially expressed between and within different tissues, Dsg2 and Dsc2a,b are common to all desmosomal tissues. In screening a panel of bladder carcinoma cell lines for expression of these desmosomal components, it is of note that both proteins are absent or expressed at low levels in invasive cell lines displaying minimal plakoglobin protein. Indeed, reduced desmosome formation in invasive bladder tumours has been reported (Alroy *et al*, 1981). Restoration of plakoglobin to EJ cells resulted in increased detection of desmoglein although we did not determine whether this represents transcriptional upregulation or reduced turnover of protein possibly complexed with plakoglobin. Restoration of desmosome formation following introduction of plakoglobin has been reported in a squamous carcinoma cell line (Parker *et al*, 1998) and desmosomal adhesion has been shown to inhibit invasion (Tselepis *et al*, 1998). However, the absence of detectable desmoglein or desmocollin in J82 cells suggests that the inhibition of migration recorded in J82 plakoglobin transfectants is not dependent upon plakoglobin interactions with desmosomal proteins.

The signalling mechanism(s) by which plakoglobin exerts its tumour suppressor activity in bladder cells remains unclear. In numerous human cancers, the wnt/*β*-catenin signalling pathway is constitutively activated eliciting activation of specific target genes including c-myc (He *et al*, 1998; Kolligs *et al*, 2000). Winn *et al*, (2002) showed that re-expression of plakoglobin in non-small-cell lung carcinoma cell lines reduced Tcf activity. Our results also showed that restoration of plakoglobin into bladder carcinoma cells reduced TOPFLASH activity but FOPFLASH activity was also reduced, suggesting the effect was not a wnt specific event.

Our observations support the work of Thievessen *et al* (2003), who demonstrated that the wnt/*β*-catenin signalling pathway is inactive and appears to be turned off in urothelial cancers. The basal wnt activity in both the EJ and J82 cell lines was comparable to that seen in the urothelial cell lines in the Thievessen study, suggesting that the wnt pathway was not activated in these lines. When *β*-catenin was added to the assay, J82 showed minimal activation whereas EJ showed a modest increase, which was comparable to that reported for the bladder cell line SW1710 (Thievessen *et al*, 2003). The bladder cells we have used in this study express N-cadherin in the absence of E-cadherin; therefore, our results support the idea that E-cadherin expression is not the only factor determining inducibility of wnt signalling in urothelial cancer.

In keratinocytes, tyrosine phosphorylation of plakoglobin results in its translocation from the cell membrane to the nucleus where it binds to nuclear Tcf/Lef (Hu *et al*, 2003). In addition, nuclear plakoglobin expression has been detected in renal cell carcinomas, where it was strongly associated with clear cell type and high grade (Aaltomaa *et al*, 2004). In this study, immunohistochemistry revealed the presence of plakoglobin in the cytoplasm and at the membrane but not in the nucleus of EJ and J82 transfectants. The absence of nuclear plakoglobin in transfectants may be indicative of a lack of detectable wnt activity in the bladder model.

We found no significant change in c-myc protein levels following forced expression of plakoglobin in bladder cell lines EJ and J82. In addition, we observed no change in the activation status of MAP kinase or Akt in plakoglobin transfectants even though activation of Akt has been implicated in bladder cell invasion. Hence, plakoglobin in bladder carcinoma cells must be exerting a tumour suppressor role via alternative signalling pathways from those established in other carcinoma cell models.

In summary, the restoration of plakoglobin expression in N-cadherin expressing bladder carcinoma cell lines reduced the migratory capacity of the cells, reduced growth in soft agar and suppressed the tumorigenic potential of the cells. These results demonstrate that plakoglobin acts as a tumour suppressor gene in bladder carcinoma cells and that the silencing of plakoglobin gene expression is an important event in bladder tumour progression.
